# Review of sEMG for Exoskeleton Robots: Motion Intention Recognition Techniques and Applications

**DOI:** 10.3390/s25082448

**Published:** 2025-04-13

**Authors:** Xu Zhang, Yonggang Qu, Gang Zhang, Zhiqiang Wang, Changbing Chen, Xin Xu

**Affiliations:** 1 Shendong Coal Group Co., Ltd., CHN Energy Group, Yulin 017209, China; zhangxu_chnenergy@163.com (X.Z.); quyonggang0204@163.com (Y.Q.); zhanggang_chnene@163.com (G.Z.); wangzhiqiang_chnen@163.com (Z.W.); 2 The Research Center for Mine Ventilation Safety and Occupational Health Protection of the State Energy Group, Yulin 017209, China; 3 China Coal Research Institute, Beijing 100013, China; xuxinucas@163.com; 4 State Key Laboratory of Intelligent Coal Mining and Strata Control, Beijing 100013, China

**Keywords:** surface EMG, exoskeleton robot, intention recognition, human–robot interface, rehabilitation robotics, deep learning, human–machine fusion-embodied intelligence

## Abstract

The global aging trend is becoming increasingly severe, and the demand for life assistance and medical rehabilitation for frail and disabled elderly people is growing. As the best solution for assisting limb movement, guiding limb rehabilitation, and enhancing limb strength, exoskeleton robots are becoming the focus of attention from all walks of life. This paper reviews the progress of research on upper limb exoskeleton robots, sEMG technology, and intention recognition technology. It analyzes the literature using keyword clustering analysis and comprehensively discusses the application of sEMG technology, deep learning methods, and machine learning methods in the process of human movement intention recognition by exoskeleton robots. It is proposed that the focus of current research is to find algorithms with strong adaptability and high classification accuracy. Finally, traditional machine learning and deep learning algorithms are discussed, and future research directions are proposed, such as using a deep learning algorithm based on multi-information fusion to fuse EEG signals, electromyographic signals, and basic reference signals. A model with stronger generalization ability is obtained after training, thereby improving the accuracy of human movement intention recognition based on sEMG technology, which provides important support for the realization of human–machine fusion-embodied intelligence of exoskeleton robots.

## 1. Introduction

The problem of aging is very serious in today’s world. In developed countries, aging has become an important factor restricting economic development. At the same time, it also brings huge challenges to the fields of elderly care, medical care, rehabilitation, etc. Especially in the medical field, the decline of motor function in the elderly brings great risks to personal health and may also aggravate cardiovascular or nervous system diseases and even leave serious sequelae [1,2,3]. In addition, the impairment of physical function caused by illness or old age will also seriously affect the patient’s ability to work independently, as well as their quality of life, which causes serious psychological or mental illness.

Generally, life assistance measures for the frail population mainly include manual care, motion assistance devices, and exoskeleton assistance. Manual care is costly, and the effectiveness and labor occupation are difficult to apply proportionally to rehabilitation. Motion assistance devices, such as wheelchairs, crutches, and braces, have single functions and can only help patients complete basic movements. They are also difficult to carry and effectively utilize to help disabled people lead a normal life [4,5,6,7].

Due to the complex structure of the human body and joints, it is difficult for traditional assistive products to meet these needs, so the development of exoskeleton robots is extremely important and urgent. Exoskeleton robots are assistive machines that can be worn on the outside of the human body to provide additional strength. As an emerging technology, combined with advanced perception and control strategies, exoskeletons can provide precise and stable motion assistance. In heavy industry, manufacturing, and logistics, workers wearing waist exoskeletons can minimize lumbar spine injuries; in the field of sports rehabilitation, injured people can use exoskeleton robots to assist in limb rehabilitation; in the fields of military, firefighting, and mine safety, wearing exoskeletons can greatly enhance the combat capability of individual soldiers. Currently, exoskeleton robots have become a hot topic in the field of robotics research [8].

Exoskeleton robot research is divided into two categories: upper-limb exoskeletons and lower-limb exoskeletons. Upper-limb exoskeleton robots are mainly aimed at the upper limbs and joints of the human body, including shoulders, elbows, wrists, and fingers, while lower-limb exoskeleton robots are mainly aimed at the lower limbs and joints, including hips, knees, ankles, etc. The lower limbs mainly complete actions such as walking and squatting, while the upper limbs are more complex and diverse, completing various posture changes according to the actual working situation. Due to the large differences in the work tasks and structural functions of the upper and lower limbs, the corresponding upper and lower-limb exoskeleton robots also differ in structure, functional design, control strategy, etc. [9,10,11]. This article will focus on upper-limb exoskeleton robots.

Usually, upper-limb exoskeleton robots are composed of sub-modules, such as a perception system, control system, and decision system. The perception system is used to measure human physiological signals or physical signals, and the decision system processes and analyzes the signals to obtain continuous human movement intentions. Finally, the control system outputs a reasonable amount of control to make the drive device take corresponding actions to assist the user in exercising [10,12,13].

However, existing upper-limb exoskeleton robots face great challenges in both software and hardware. The inability to fully predict or identify the user’s movement intention results in the control system being unable to respond quickly, timely, and accurately, resulting in low power efficiency and even adverse effects on the user, greatly limiting the user’s interactive control performance. Therefore, accurately capturing movement intention information and judging movement intention are crucial for upper-limb exoskeleton interactive control. The interactive control method of the exoskeleton robot must find an appropriate input signal to accurately provide movement intention information with less delay. Movement intention signals are divided into two categories: physical signals and human biological signals [14,15,16,17,18,19].

Physical signals refer to signals from angle sensors, force sensors, acceleration sensors, etc., which are usually measured using inertial sensors (IMU) [20,21,22,23,24,25], plantar pressure sensors [26,27,28,29], etc. With the rapid development of sensor technology, these signals have high accuracy and reliability, and can well reflect the user’s movement process, and the drive, control, and communication technologies are relatively mature and are often used in various control fields. However, the change of this signal can only occur after the action occurs, and after the delay of the perception, control, and drive systems, the output to the end controller may cause excessive delay.

Human biological signals include electromyographic signals (EMG), electroencephalographic signals (EEG) [30,31], electrooculographic signals (EOG) [32,33], etc. Bioelectric signals are the potentials and signals excited when neurons carrying behavioral information are transmitted to related tissues/organs. They do not depend on the physical movements performed by the limbs. The signals have good real-time performance and can well balance the relationship between the initial movement intention and the interpretability of the signals. By processing the signals, the current state and movement trend can be obtained. With the development of neuroscience, the human biological signals that are currently widely used in intention recognition are surface electromyographic signals (sEMG). More and more exoskeleton robots use EMG signals as input signals to collect and record the current human movement state.

Therefore, this paper will review the research progress on exoskeleton robots, sEMG technology, and intention recognition technology. Then, keyword clustering analysis is used to comprehensively analyze the relevant literature. The work in this paper will fill the gap in the field of exoskeleton robots and the review of human motion intention recognition using sEMG technology and provide important support for the realization of human–machine fusion and embodied intelligence of exoskeleton robots.

## 2. Upper Limb Exoskeleton

**Figure 1:**
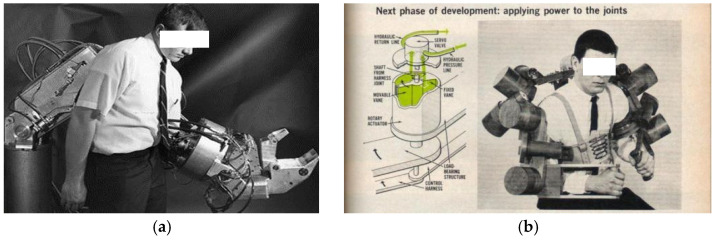
Schematic diagram of the ‘Hardiman’ exoskeleton (**a**) and ‘Man Amplifier’ exoskeleton (**b**).

The development of upper limb exoskeleton robots can be divided into three stages: early exploration stage, technology accumulation stage, and rapid development stage. Early exploration stage: 1960s–1980s, represented by Cornell Aeronautical Laboratory’s “Man Amplifier” and General Electric’s “Hardiman”. The “Hardiman” upper limb exoskeleton was developed by General Electric in the United States [34,35], as shown in Figure 1a. It is driven by hydraulics and electricity and can amplify the wearer’s strength by 25 times. The project provided valuable experience for subsequent exoskeleton research and design. At the same time, the Cornell Aeronautical Laboratory (CAL) in the United States also launched the Man Amplifier model upper limb exoskeleton robot, as shown in Figure 1b, which is mainly driven by electricity and aims to enhance human upper limb strength and endurance through mechanical structure [36].

**Figure 2:**
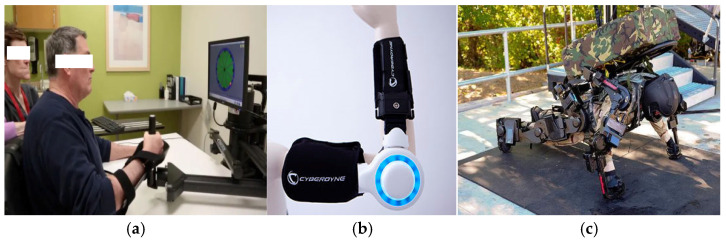
Schematic diagram of the ‘MIT-Manus’ exoskeleton (**a**), ‘HAL’ exoskeleton (**b**), and ‘XOS’ exoskeleton (**c**).

Technology accumulation stage: 1990s–2000s. MIT-Manus is the earliest upper limb exoskeleton robot used for rehabilitation training. It can accurately interact with patients and provide support when patients need it. At the same time, it can combine virtual reality technology to provide rich visual input, assist sensory motor training, and increase the fun and challenge of upper limb functional training [37], as shown in Figure 2a.

The HAL (hybrid assistive limb) exoskeleton developed by Cyberdyne of Japan mainly adopts the design method of a motor–reducer–exoskeleton mechanism. It can automatically adjust the size of the device’s assistance according to the human body’s movement intention. Its main use is for elderly care, disabled assistance, firefighting, police, and other dangerous operations [38], as shown in Figure 2b.

The “XOS” exoskeleton developed by Sarcos of the United States is mainly for specific application scenarios of material handling. Through its innovative soft structure and ultra-light material design, it can significantly enhance the strength and endurance of soldiers; it can be used to carry heavy objects and perform high-intensity tasks [39], as shown in Figure 2c.

**Figure 3:**
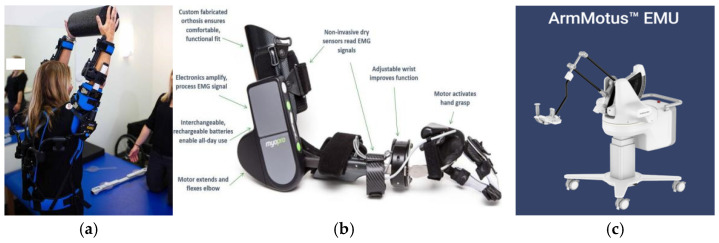
Schematic diagram of the ‘EksoUE’ exoskeleton (**a**), ‘MyoPro’ exoskeleton (**b**), and ‘Fourier Arm’ exoskeleton (**c**).

Rapid development stage: 2010s–present, EksoUE is a wearable upper limb exoskeleton that uses motors, springs, and mechanical structures for flexible driving. Its main function is to help patients with injured shoulders and arms perform guided, supportive movements and training during rehabilitation [40], as shown in Figure 3a.

MyoPro is an electric arm that uses sEMG technology to read weak nerve signals on the surface of the skin, thereby helping the wearer restore paralyzed or weak upper limb functions and achieve autonomous operation in daily life [41], as shown in Figure 3b.

The “Fourier Arm”, developed by China’s Fourier Intelligence Company, is a lightweight end-traction upper limb rehabilitation exoskeleton robot that uses artificial intelligence and virtual reality technology to provide personalized rehabilitation training programs, as shown in Figure 3c.

In summary, upper limb exoskeleton robots have a wide range of applications. At the same time, with the rapid development of mechanical technology, sensor technology, material technology, and control technology, exoskeleton products are becoming increasingly mature and gradually moving towards practical applications. They are used to assist patients with muscular atrophy symptoms in their daily lives, for rehabilitation of stroke and hemiplegia patients, and for enhancing human functions.

## 3. Surface Electromyography

### 3.1. Evolution of Surface Electromyography Technology

Electromyography (EMG) reads signals generated by skeletal muscle activity, which comes from the potential generated by the activation of motor units. Surface electromyography (sEMG) reads the electrical signals generated by the muscle and nerve activity in the superficial layer of the epidermis. It is the sum of a large number of asynchronous action potentials of muscle fibers, which can directly reflect muscle activity. When the muscles are relaxed or tense, the force generated and the amplitude of the electromyographic signal have an approximately linear correspondence [42,43,44,45].

The development of surface electromyography (sEMG) technology can be traced back to the early 18th century, when Professor Galvani discovered the biological discharge phenomenon produced by frog thigh muscles during exercise and announced the theory of “animal electricity”, proving that muscle contraction has a strong relationship with electromyography. From then on, humans opened a new chapter in understanding electromyography [46].

Professor Willem Einthoven invented the string galvanometer in 1895 to record weak action potential signals. It was first used in clinical medicine to analyze and record electrocardiogram signals. This technology provided a theoretical basis for electrophysiology and pointed out the direction for the subsequent interpretation of electromyographic signals [47].

In 1922, Professor Joseph Erlanger used a cathode ray oscilloscope to replace the traditional galvanometer to successfully record the electromyographic signal for the first time, accurately recording the action potential of nerve fibers, revealing that nerve fibers control muscle contraction through electrical signals, and providing a theoretical basis for the study of electromyographic signals [48].

**Figure 4:**
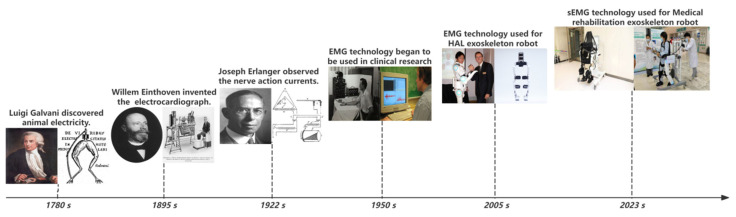
Evolution diagram of surface electromyography signal technology and application.

Since 1950, EMG technology has been widely used in clinical medical diagnosis and research of diseases such as muscle weakness and muscular atrophy, providing important reference information for doctors and patients. In 2005, the first HAL exoskeleton robot equipped with electromyography technology was launched in Japan, marking the beginning of the application of electromyography technology in the field of human–computer interaction. With the development of integrated circuits, flexible circuit boards, and microelectronics technology, electromyography technology has gradually developed towards non-invasive surface electromyography technology that can monitor muscle activity in real time. Combining sEMG technology with exoskeleton rehabilitation robots can achieve real-time monitoring and feedback of patients’ muscle activity, providing more accurate guidance for rehabilitation training. In 2023, medical rehabilitation exoskeleton robots equipped with surface electromyography technology were put into use in hospitals in Zhejiang, China. Due to its excellent rehabilitation treatment effect, it has won unanimous praise from patients. The evolution of electromyography technology is shown in Figure 4 [49].

### 3.2. Introduction to Surface EMG Signal Acquisition Technology

**Figure 5:**
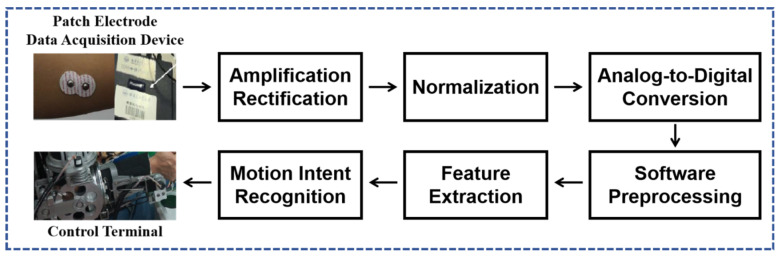
EMG signal acquisition and processing flowchart.

Myoelectric signals are weak signals and are very susceptible to external interference. The noise mainly includes several aspects: the noise generated by the muscles during upper limb movement, the noise generated by the relative movement between the electrode patch and the muscle, environmental noise, electrical noise, ECG artifacts, etc. Special weak signal processing methods are required [50]. The EMG signal acquisition and processing flow are shown in Figure 5.

Generally speaking, surface electromyographic signals have the following characteristics:

(1) Small amplitude, slight change, and susceptible to interference: the amplitude is generally 100–2000 μv, with a maximum of no more than 5 mv and a root mean square of 0–1.5 mv. Because the equipment collects data with high accuracy, it is very susceptible to environmental noise, and the data quality of different hardware is also different, so the performance and stability of the collection equipment are required to be high.
(2) Low-frequency characteristics: The frequency concentration area of surface electromyographic signals is 10–500 Hz, and the energy is concentrated at 30–150 Hz. Even if the muscle force changes, the frequency distribution of the electromyographic signal remains relatively stable.
(3) Amplitude alternation: The amplitude of the surface electromyographic signal can be positive or negative, and the absolute value of the signal has an approximate proportional relationship with the muscle force.
(4) Pre-emptive nature: Since the signals transmitted by the nervous system of the human body during movement are transmitted to the arm through the central nervous system, the electromyographic signal can already reflect the movement of the muscle before the arm moves. The change in the electromyographic signal will be ahead of the change in human body movement, which is proactive [51].

These weak signals are amplified, filtered, normalized, etc., to remove noise and interference. After AD conversion, they are finally converted into discrete electromyographic signals that can be analyzed. The collected signals reflect the activity status of the nerves and muscles [52]. Noise is difficult to prevent during the acquisition of electromyographic signals, so after the original signal is acquired, signal preprocessing is required to remove noise and lay the foundation for subsequent discrimination and control.

In order to remove high-frequency noise in sEMG signals, the passband frequency is set within the energy concentration range, and a Butterworth bandpass filter is usually applied for filtering. Then, the software algorithm is used to remove erroneous values or data out of range, and feature extraction can be performed. Feature extraction of sEMG signals refers to the use of low-dimensional space to represent samples by mapping (or transforming) when the number of original features is relatively large, or the samples are in a high-dimensional space [50].

Time domain feature signals include mean absolute value, root mean square, mean, integrated EMG value, maximum and minimum values, variance, standard deviation, histogram, mean absolute value, number of zero crossings, skewness, kurtosis, Willison amplitude, and time domain model parameters, similar to AR model parameters to indicate the role of sEMG in the motion estimation process [53,54,55,56,57].

Common frequency domain features include conventional spectrum based on Fourier transform, median frequency, average frequency, cepstrum analysis, spectrum analysis based on parameter model, etc. Common time–frequency domain features include short-time Fourier transform, wavelet transform, wavelet packet transform, high-order spectrum analysis, Wigner–Ville distribution, etc. [58].

After the acquisition, preprocessing, and feature extraction are completed, the extracted feature matrix needs to be used for motion intention recognition. Different classification algorithms will select different features for prediction. When using it, it is necessary to flexibly select feature combinations according to actual conditions.

### 3.3. Keyword Cluster Analysis

**Figure 6:**
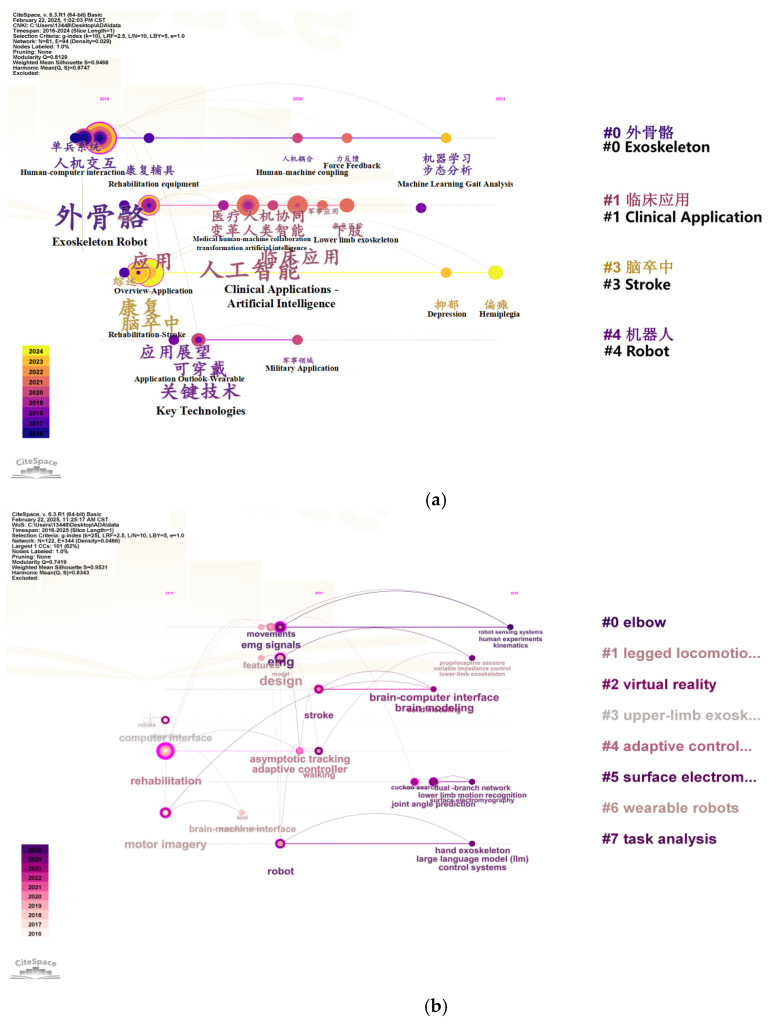
Using CiteSpace to plot the timeline with Motion Intention Recognition, Exoskeleton, and Scenario as the keywords. (**a**) China National Knowledge network document mapping. (**b**) Web of Science literature mapping.

In order to more comprehensively and accurately grasp the application scenarios of sEMG technology, motion intention recognition technology, and exoskeleton robots, CiteSpace (CiteSpace 6. 3. R1. (64-bit) Basic) was used to analyze 128 Chinese and foreign scientific research papers with keywords focused on Motion Intention Recognition, Exoskeleton, and Scenario, as shown in Figure 6.

CiteSpace provides two important indicators for judging the effect of graph drawing based on the network structure and the clarity of clustering, namely modularity Q (Q value for short) and mean silhouette (S value for short). Among them, the Q value is generally in the interval [0, 1]. If Q > 0.3, it means that the view structure is significant; if S > 0.5, the clustering is considered reasonable; when the S value is 0.7, the clustering is considered to be efficient and convincing. As shown in Figure 6, the Q values of the two visualizations are 0.8129 and 0.7419, both greater than 0.3; the S values are 0.9468 and 0.9531, both greater than 0.7. The above analysis shows that the clustering structure of Chinese and English literature analyzed using the keywords intent recognition, exoskeleton, and application scenarios is significant and reasonable [59]. The criteria for literature screening in this article are academic papers related to the keywords of this article, selected from the Web of Science and CNKI official website, covering the time range of nearly ten years.

By using the keywords Motion Intention Recognition, Exoskeleton, and Scenario to analyze the significance and rationality of the clustering structure of Chinese and English literature, it can be concluded that exoskeleton robots using intention recognition technology have gradually become a hot topic in social research in recent years, the research direction is mainly focused on exoskeleton robots using intention recognition technology, but the application scenarios in China are relatively singular, mainly rehabilitation medicine, such as rehabilitation training for stroke patients. Intention recognition technology is more advanced in China than in foreign countries, and it is the first to introduce artificial intelligence to recognize human movement intentions. Foreign application scenarios, intention recognition technology, and application carriers are more diverse, and their main application scenarios are virtual reality, rehabilitation medicine, adaptive training, wearable robots, human motion analysis, upper-limb assistance, etc. Intention recognition technology innovatively uses a human–computer interface to achieve it.

Table 1 summarizes the latest innovative achievements of other articles using intention recognition technology on exoskeleton robots, such as artificial intelligence technology, human–computer interface technology, surface electromyography signal technology, etc.

**Figure 7:**
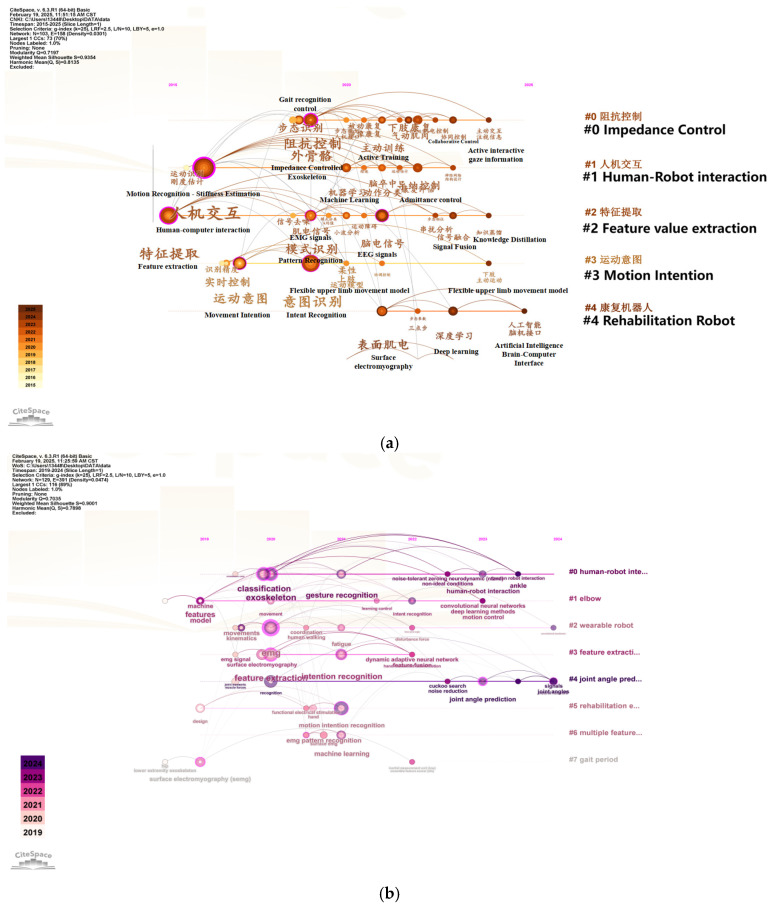
Using CiteSpace to analyze 321 Chinese and foreign scientific research papers in which keywords focus on sEMG, Motion Intention Recognition, and Exoskeleton. (**a**) China National Knowledge network document mapping. (**b**) Web of Science literature mapping.

In order to further understand the importance, relevance and related algorithms of sEMG technology in the process of human motion intention recognition by exoskeleton robots, 321 Chinese and foreign scientific research papers were analyzed using CiteSpace software, with the keywords mainly focusing on sEMG, Motion Intention Recognition, and Exoskeleton, and the results are shown in Figure 7.

The CNKI visualization Q value obtained by CiteSpace analysis is 0.7197, and the S value is 0.9354. The Web of Science visualization Q value is 0.7035, and the S value is 0.9001. The clustering is significant and reasonable. Through analysis, it can be concluded that sEMG technology is important and highly relevant in the process of human motion intention recognition by exoskeleton robots. The algorithms applied are mainly machine learning and deep learning algorithms. Table 2 summarizes the latest innovative results of the remaining articles in using sEMG technology for motion intention recognition.

## 4. Movement Intention Recognition Technology Based on Surface Electromyography Signal

Movement intention classification refers to the recognition of the intention of upper limb movement, which usually includes three types: discrete action recognition, continuous action recognition, and continuous motion parameter recognition. The classification and recognition of discrete and continuous actions mainly classify preset actions. According to the data and prediction model, the most likely action is output, such as shoulder or elbow extension, and an independent action is output as a prediction of the user’s upcoming action. Most classification methods are based on artificial intelligence methods. Feature extraction is required before intention recognition. The extracted features need to be used for movement intention recognition using a classifier, and then the classification or regression results are converted into driving commands provided to the exoskeleton. The most commonly used classifiers include Bayesian classifiers [76], support vector machines [77], BP neural networks [78], linear discriminant analysis [79], fuzzy logic [80], random forests [81], etc. These methods learn rules from previous data and labeled training data sets, and the recognition accuracy of the classifier is the percentage of correctly output actions [82].

Continuous motion parameter recognition can ensure the continuous matching of human–machine motion. Compared with motion recognition, motion parameter recognition is more comprehensive and can effectively supplement the problem of incomplete motion classification. These motion parameters include joint angle, torque, acceleration, angular velocity, etc., which are usually obtained by regression. Compared with discrete motion intention classification, continuous motion intention recognition can adjust and update the trajectory online in real time, has higher flexibility, can handle the interference caused by emergencies in a timely manner, and can achieve better human–machine interaction.

**Figure 8:**
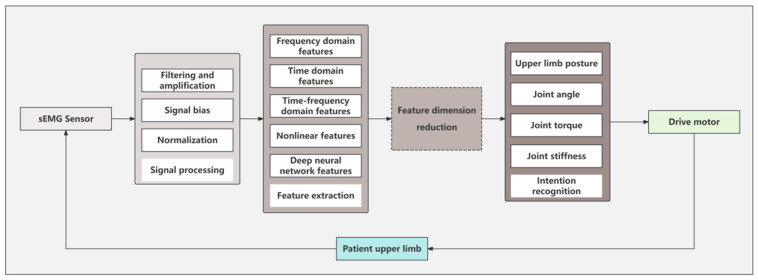
Flowchart of motion intention recognition algorithm based on artificial intelligence algorithm.

Because the surface electromyography signal has a low signal-to-noise ratio and certain randomness, it is a common processing method to first process the surface electromyography signal, extract features, and then perform motion intention recognition based on artificial intelligence algorithms. The processing flow is shown in Figure 8. The artificial intelligence algorithms used for final intention discrimination include traditional machine learning algorithms and deep learning algorithms. Machine learning requires less data and uses simple models and fewer parameters to simulate and learn. Deep learning uses a large number of layers and nonlinear relationships to simulate complex relationships in nature.

By definition, deep learning is a special method and extension of machine learning. Different algorithms perform differently on different data sets. There are many reasons behind this phenomenon. The classification accuracy is not only affected by the number of categories in the classification task, the number of channels of the electromyographic signal, the acquisition time, the quality of the sensor, the layout of the sensor, the muscle fatigue state, but also the user’s health status, the posture of other parts of the body, the speed of limb movement, the mutual interference and noise between electrodes, and even the ambient temperature. These factors will have a serious impact on the classification. Therefore, finding an algorithm with strong adaptability and high classification accuracy has become the focus of current research. In order to facilitate the distinction, traditional machine learning and deep learning algorithms are used separately to better highlight the advantages and applicable conditions of different methods.

## 5. Movement Intention Recognition Based on Traditional Machine Learning

Traditional machine learning models have been maturely applied in the field of sEMG-based action recognition research, such as linear discriminant analysis (LDA), K-nearest neighbor classification (kNN), support vector machine (SVM), random forest, naive Bayes, etc. By learning from the training data, a parameterized model is obtained, and the model is used for prediction and decision-making. The trained model has high classification accuracy and strong generalization ability, but the feature selection is random and mainly relies on manual experience. It has limitations in adaptability, and the recognition results are easily affected.

In recent years, in order to eliminate the adverse effects of environmental changes on recognition accuracy, many experts have focused on how to improve the practicality of the algorithm without reducing the accuracy.

Duan et al. [83] designed a gesture recognition system that uses linear discriminant analysis (LDA) combined with time domain features to achieve the classification of nine gestures using sEMG signals from three channels. By applying root mean square ratio (RMSR) and autoregression (AR) as key features, the influence of force changes is effectively eliminated. A high average accuracy of 91.7% was achieved on a dataset of eight subjects, demonstrating its high efficiency and accuracy in gesture recognition. In addition, Qi et al. [84] proposed a method to use linear discriminant analysis (LDA) and extreme learning machine (ELM) to reduce redundant information in sEMG signals and improve recognition efficiency and accuracy. The characteristic graph slope (CMS) was innovatively extracted to strengthen the relationship between features across time domains and enhance the feasibility of cross-time recognition. This recognition framework can improve the generalization ability of human–computer interaction in long-term use and simplify the data collection stage before daily use of the device.

Naik [85] proposed a method for finger extension and flexion classification using a modified independent component analysis (ICA) weight matrix. First, the data were processed by principal component analysis (PCA), and the ICA matrix ratio was customized for different individuals for separation. The extracted features were classified by linear discriminant analysis (LDA), achieving a classification accuracy of nearly 90%, fully considering individual differences, and greatly improving the efficiency of the algorithm. Ali et al. [86] proposed a hierarchical control framework based on sEMG. Through machine learning methods, sEMG signals were analyzed in real time to achieve active–passive exoskeleton control for human–machine collaboration. When applied to exoskeleton robots, it can realize functions such as intention recognition, force regulation, and control mode regulation.

Since the classification decision boundary of upper-limb action recognition is very complex, the non-parametric method KNN classification method has shown better results. KNN is a supervised learning algorithm for classification and regression tasks. Its principle is to identify the k closest data points to a given input and classify the input according to the majority of classes among these neighbors. It mainly achieves action recognition by considering the similarity between sEMG data and samples in the training set.

Benalcázar et al. [87] proposed a real-time gesture recognition model based on the Myo armband, which learns to recognize hand movements through training. sEMG signals are used in combination with the kNN algorithm for classification, and muscle activity detection is integrated to improve processing speed and recognition accuracy. It was tested on five predefined gestures of the Myo armband, and an accuracy of 89.5% was achieved.

The action recognition method based on KNN is greatly affected by feature selection. Narayan [87] used a kNN classifier to classify sEMG signals in combination with the frequency domain (FD) and the time–frequency domain (TFD) features. Feature vectors were extracted by discrete wavelet transform. The experimental results show that the classification accuracy of the combination of TFD feature vector and kNN reaches 95.5%, which is better than 89% of the FD feature. However, the TFD feature is not always better than the FD feature for all systems. This needs to be adjusted according to system parameters and training data. Before using kNN for action recognition, appropriate features should be selected through experiments to improve the reliability of the algorithm [88].

In addition to the need to select appropriate features, environmental interference, gender, body state, etc. will affect the recognition accuracy. Bergil et al. [89] detected six basic hand movements based on the kNN algorithm and focused on the impact of gender on classification performance. The system extracts signal features through a four-layer symmetric wavelet transform and classifies and clusters after PCA dimensionality reduction. Tested on six subjects (three females and three males), a high accuracy of 86.33% to 100% was achieved.

Xue et al. [90] considered the impact of the use environment and proposed an algorithm based on tensor decomposition for hand gesture recognition in underwater environments. The algorithm uses Tucker tensor decomposition to extract and recognize the signal features of underwater hand gestures. The research team selected seven subjects to complete four hand gestures underwater, generated three-dimensional tensors through wavelet transform, and extracted signal features using tensor decomposition technology. The kNN algorithm was used for recognition, achieving a recognition accuracy of 96.43%. The KNN model is simple and easy to use. After selecting appropriate features, it has high recognition accuracy for different environments, genders, etc. However, the model needs to calculate all data when classifying, which has high computational costs and poor real-time performance. In the process of exoskeleton assistance, higher real-time performance means that the controller can change the output of the control quantity faster and achieve rapid response. Therefore, it is necessary to explore an algorithm model that can predict in real time. Support vector machine (SVM) provides a robust classification method that performs well in processing high-dimensional data and can achieve high classification accuracy even with limited training samples. It has a good real-time prediction effect and provides a new idea for the recognition of complex actions.

The SVM model processes nonlinear data through kernel functions and maps them to high-dimensional space, thereby achieving effective classification and solving nonlinear problems. The optimization of hyperparameters, namely the optimization of regularization parameters and kernel parameters, is crucial. Chen et al. [91] proposed a digital gesture pattern recognition method based on a 4-channel wireless surface electromyography signal system. The effects of three popular feature types (time domain feature (TD), autocorrelation and cross-correlation coefficients (ACCCs), spectral power amplitude (SPM)) and four popular classification algorithms (k-nearest neighbor (k-NN), linear discriminant analysis (LDA), quadratic discriminant analysis (QDA), and support vector machine (SVM)) in offline recognition were studied. Multi-kernel learning SVM (MKL-SVM) was further proposed. Experimental results show that the MKL-SVM method combined with three features achieved a maximum accuracy of 97.93%. It can be seen that the multi-kernel mapping method can significantly improve the accuracy of the SVM algorithm.

In order to better solve the real-time problem in exoskeleton control, Kong et al. [92] proposed a joint angle prediction model based on LSSVM to obtain the patient’s movement intention. According to the physiological characteristics of sEMG, preprocessing and feature extraction are completed. To address the time delay problem in real-time processing, feature optimization of time difference compensation is introduced. Experiments were conducted using sEMG signal data from six volunteers, and the results showed that this method can significantly improve the effect of rehabilitation training.

Pourmokhtari et al. developed a gesture recognition method based on single-channel EMG signals. Using kNN classification technology, five different finger movements were classified. The study used time domain features, such as maximum value (Max), minimum value (Min), and mean absolute value (MAV), combined with root mean square (RMS) and simple square integral (SSI) for classification. The results showed that the classification accuracy of the combination of MAV, Max, and Min on the four channels was 91.0%, 89.9%, 89.8%, and 96.0%, respectively, highlighting the simplicity, practicality, and cost-effectiveness of single-channel sEMG analysis [93].

Traditional machine learning algorithms have made great progress in motion intention recognition. By selecting different feature combinations, they have achieved an accuracy of nearly 90% or more on the corresponding data sets. However, feature selection and parameter setting rely on manual experience and require actual data as support, so there are still certain limitations in practical applications. A comparison of different algorithms is shown in Table 3. Deep learning relies on powerful neural networks to learn features from raw data and minimize human intervention.

## 6. Motion Intention Recognition Based on Deep Learning

Deep learning imitates and learns the working mechanism of the human brain by building multi-layer neural networks to process complex patterns and data, especially nonlinear problems that are difficult to handle with traditional machine learning methods. In addition, deep learning can automatically extract and learn features, optimize models, and process large-scale data sets, thereby improving the accuracy of predictions.

**Figure 9:**
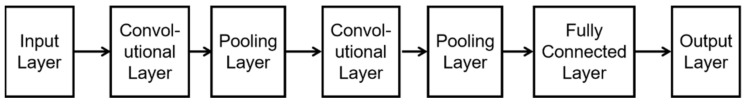
CNN architecture diagram.

sEMG-based recognition systems face challenges such as low data variability and repeatability. These problems can be effectively solved through deep learning technology. With the continuous improvement of deep learning algorithms and computing power, deep learning models have shown broad application prospects in the field of motion recognition. Deep learning networks include an input layer, multiple hidden layers, and an output layer. There is no need to manually select features to learn high-level features of input sEMG data. Common classifiers include artificial neural network (ANN), convolutional neural network (CNN) as shown in Figure 9, hidden Markov model (HMM), and long short-term memory (LSTM).

In the deep learning network structure, the CNN network can automatically learn the characteristics of hand electromyographic signals from a large amount of data and classify them. It is one of the most widely used classification networks for hand movement recognition. Tam et al. proposed a real-time control strategy for prosthetic hands based on high-density electromyography and CNN. The strategy uses deep learning and transfer learning to adapt to the unique electromyographic signal pattern of each user. Through the transfer learning method, the training time is significantly reduced, making the system installation and calibration process easier. In real-time tests on 12 healthy users, the system achieved an accuracy of up to 93.43% and a response time of less than 116 milliseconds [94].

However, in the field of exoskeletons, surface electromyographic signals (sEMG) are easily blocked by skin, fat, and other muscles and are interfered with by sweat, movement, ECG artifacts, etc. Surface electromyographic signals have a low signal-to-noise ratio and a certain degree of randomness. Lin et al. [95] proposed a high-density EMG recognition framework combining generative adversarial networks (GANs) and CNNs to target contact artifacts and loose contact interference. The framework generates synthetic signals through GANs to simulate disturbed recording conditions and uses CNNs to train these data to enhance the model’s robustness to actual signal interference. Experimental results show that the classification accuracy of this method is improved from 64% to 99% in the presence of contact artifacts and loose contact interference.

Rehman et al. [96] directly used the EMG raw signal as the input of the deep network and used the network’s intrinsic feature extraction capability for classification. The MYO armband was used as a wearable EMG sensor, and EMG data from seven healthy subjects were collected for 15 consecutive days. The use of data-driven feature extraction methods may overcome the problems of feature calibration and selection in electromyographic control. The algorithm showed lower classification error, better performance and robustness, and better adaptability to capture the time-varying nature of electromyographic signals.

In addition, factors such as different individuals and different speeds will also have a significant impact on classification accuracy. Jiang et al. [97] used a CNN structure to process sEMG signals from shoulder and upper-limb muscles to identify upper arm movement patterns, including rest, drinking, forward and backward movement, and abduction. The features of the EMG signal were extracted through the time–space convolution structure of CNN, achieving high-accuracy shoulder movement pattern recognition. The accuracy of the normal movement speed CNN model in movement pattern recognition reached 97.57%.

Mohapatra et al. proposed a method based on a time–frequency domain deep neural network (TFDDNN) for the automatic recognition of gestures in multi-channel electromyography (MEMG) sensor data. The method first segmented the MEMG signal frame and calculated the average of all channel information of each frame, then applied continuous wavelet transform to obtain time–frequency representation (TFR) and finally used the deep representation learning network model for gesture recognition, with overall accuracy rates of 92.73% and 80.33%, respectively [98]. In response to key technical challenges such as non-intuitive control schemes, lack of sensory feedback, poor robustness, and single sensor modality, Jiang et al. [99] proposed a biorobotics research method for non-invasive myoelectric neural interface for upper limb prosthesis control. Deep learning combined with sEMG signal decomposition was used to improve the intuitiveness, naturalness, and user acceptance of prosthetic control. At the same time, since the myoelectric signals of amputees are different from those of ordinary people, using amputees’ myoelectric signals to accurately identify multi-force gestures is of great significance for amputees. Triwiyanto et al. [100] also proposed a classification method based on deep neural networks, which used amputees’ electromyographic signals to improve the recognition accuracy of multi-force gestures. It can recognize six gestures and maintain robustness at different force levels, with an average accuracy of 92.0%. This method can be applied to the development of prosthetic hands for amputees based on modern high-speed embedded system technology, and the algorithm can be applied to actual control.

At present, although a large number of studies have investigated the real-time control of upper limb exoskeletons based on user intentions and specific motion trajectories, the prediction of body joint position based on myoelectric features is still less explored. Sedighi et al. [101] proposed a new method to generate exoskeleton joint trajectories using a convolutional neural network and a long short-term memory (CNN-LSTM) model to effectively interpret the user’s usage intention. This method predicts the user’s intended position 450 ms ahead of the user by exploiting the natural delay in electromyographic activity.

Lv et al. proposed an algorithm combining a self-organizing map (SOM) and radial basis function (RBF) network to identify hand movement intention from sEMG signals. SOM was used for feature selection and preliminary clustering, followed by principal component analysis (PCA) for dimensionality reduction, and finally, the RBF network was used for pattern classification. A dataset of sEMG signals from six volunteers of eight gestures collected by MYO armband sensors was used. The innovation lies in the combination of SOM and RBF, which improves the accuracy and efficiency of recognition. It has the advantages of a maximum recognition rate of 100%, an average recognition accuracy of 96.875%, and a response time of 0.437 s [102].

Rajapriya et al. proposed a deep learning algorithm based on a high-order statistics-frequency domain (HOS-FD) feature set to improve the classification accuracy of hand movements in myoelectric control systems. Bispectral analysis was used to capture the nonlinear characteristics in EMG signals and combined with deep neural network (DNN) for pattern recognition. EMG datasets containing different limb positions were used for training and testing. The innovation lies in the proposal of a new HOS-FD feature set, which is translation/scale invariant and suitable for classification tasks under dynamic limb position changes. The final result presented is that when trained on all five limb position data, the DNN model achieved a high classification accuracy of 99.16% ± 0.14 [103].

Simão et al. proposed an online gesture classification algorithm based on EMG signals, which was implemented using a recurrent neural network (RNN), especially a long short-term memory network (LSTM). The EMG signals collected from the forearm muscles were used to improve the performance of online gesture classification through the dynamic model of LSTM. The UC2018 DualMyo and NinaPro DB5 datasets were used for evaluation, and a new performance indicator, gesture detection accuracy, was proposed to evaluate the performance of the model in online classification. In terms of final classification accuracy, LSTM and GRU significantly outperformed RNN and FFNN in terms of training and inference time and were similar to the static model (FFNN) in terms of accuracy, but GRU and LSTM performed slightly better [104].

However, the use of reinforcement learning (RL) technology to classify EMG is still a new and open research topic. RL-based methods have the advantages of good classification performance and online learning of user experience. Caraguay et al. [105] proposed a gesture recognition system based on deep and dual-deep Q networks. A feedforward artificial neural network (ANN) was used as a policy representation, and the performance of adding a long short-term memory (LSTM) layer was evaluated. The results showed that the DQN model without LSTM performed best in classification and recognition accuracy, reaching 90.37% and 82.52%, respectively, proving the effectiveness of reinforcement learning methods in classification and recognition problems based on EMG signals. And Table 4 gives a comprehensive comparison of deep learning model algorithms.

In summary, upper limb exoskeleton motion intention recognition technology based on deep learning has made great progress in recognition accuracy and generalization ability, but it has also put greater pressure on the demand for data and computing resources. Limited by the complexity of the model, the update and maintenance costs of the algorithm architecture have also increased. How to adapt different models with minimal overhead has also become one of the current research focuses. The comparison of some model algorithms is shown in Table 4.

Although the recognition of motion intention based on electromyographic signals has made great progress, there are still some practical problems that limit the actual application effect of the algorithm. First, electromyographic signals are very weak and easily interfered with by the external environment. They are also highly dependent on the installation location and sensor model. Second, the strength of electromyographic signals varies greatly between different people, and there are certain differences in the amplitude and frequency distribution of electromyographic signals between healthy people and patients with diseases. At present, the data sets of most models and algorithms are from healthy people, so when the models are applied to patients, the classification accuracy will be poor. In addition, electromyographic signals are also affected by many factors, such as age, fatigue, and exercise habits. Finally, although deep learning has high recognition accuracy, the recognition model is complex to calculate, requires a large amount of data calculation, and has weak model migration ability. When applied to human motion signal processing based on sEMG technology, there are obstacles in real-time and individual differences.

Therefore, some methods for improvement have been proposed, such as transfer learning methods. Chen et al. proposed a transfer learning strategy [106] to improve the generalization of sEMG-based gesture recognition and reduce the training burden. A CNN is trained to capture gesture features, and then these features are used in two target networks: pure CNN and CNN + LSTM. On three different datasets, the TL strategy significantly improved gesture recognition accuracy by 10–38%, significantly shortened training time, and ensured a recognition accuracy of more than 90%, which is of great value to the development of myoelectric control systems. Côté-Allard et al. used transfer learning technology to enhance CNN to utilize inter-user data from the first dataset and reduce the burden of data generation imposed on a single individual. On a dataset with 17 participants, the improved CNN achieved an average accuracy of 97.81% on seven hand/wrist gestures [107]. And Table 5 gives a comprehensive comparison of machine learning, deep learning, and hybrid approaches..

Multi-source information fusion can improve the adaptability of the model or algorithm, reduce the impact of noise, and improve classification accuracy [108,109,110]. EEG signals and sEMG signals are used as model inputs, and deep multi-task learning is then used to predict the exoskeleton user’s movement intentions and cognitive states. After training, a model with stronger generalization capabilities is obtained, achieving “human–machine symbiosis” control and embodied intelligence that is more in line with the human neural mechanism. At the same time, while optimizing the algorithm, we can also start from the driving method of the exoskeleton. Long Zhang et al. [111] proposed that parallel elastic actuators can reduce the joint impedance of the exoskeleton. PEA stores energy through elastic elements and reduces the peak torque of the actuator, simulating the energy circulation characteristics of human tendons, thereby optimizing the flexibility of human–computer interaction. Luo, S. et al. [112] proposed an exoskeleton machine design architecture based on multi-source information fusion of biomechanical models, robot models, and data-driven signals. To a certain extent, it can provide prior knowledge through physical models and improve the development efficiency and machine learning efficiency of exoskeleton robots. Achilli et al. [113] proposed a multi-source information fusion design concept for a hand rehabilitation exoskeleton, which achieves precise adaptation of human–machine collaboration by integrating information such as the user’s clinical injury data, hand characteristics, and exoskeleton technical characteristics (rigid/soft/hybrid architecture). Geonea et al. [114] proposed an exoskeleton robot design idea based on multi-source data fusion, which mainly integrates mechanical design data, motion simulation data, and experimental data to provide basic data for closed-loop optimization design of virtual simulation and exoskeleton experimental verification, thereby reducing the iteration cost of physical prototypes. Tian J et al. [115] proposed a shoulder exoskeleton design scheme based on multi-source data fusion, which integrates the mechanical structure design data, controller design data, and experimental test data of the exoskeleton robot for analysis, providing a reference for the applicability analysis of the exoskeleton.

## 7. Summary and Prospects

This paper introduces the research progress of upper-limb exoskeleton robots, sEMG technology, and intention recognition technology in detail, analyzes the literature using keyword clustering analysis, and comprehensively discusses the application of sEMG technology, deep learning methods, and machine learning methods in the process of human movement intention recognition by exoskeleton robots.

Since sEMG signals themselves have the characteristics of weak intensity and susceptibility to environmental interference, and the characteristics of electromyographic signals of different individuals are significantly different, a comparative analysis of movement intention recognition technology based on electromyographic signals is conducted it is concluded that it is very challenging for exoskeleton robots to use sEMG signals to recognize human movement intentions. Therefore, the current research focuses on finding algorithms with strong adaptability and high classification accuracy. This paper further explores machine learning methods and deep learning methods. Traditional machine learning methods rely on manual feature extraction in electromyographic signal processing, which is suitable for small-scale data sets but has limited generalization ability and adaptability. Deep learning-based methods can automatically learn complex features from raw data, especially performing well on large-scale data sets, and have stronger generalization and adaptability. However, in practical applications, deep learning models need to overcome the characteristics of sEMG signals, which are weak and susceptible to environmental interference, as well as the significant differences in the characteristics of electromyographic signals of different individuals and the challenges of real-time performance. Therefore, research on the use of sEMG signals for human motion intention recognition by exoskeleton robots needs to further optimize the algorithm model and fuse multi-source information. This paper proposes a deep learning algorithm based on multi-information fusion to fuse EEG signals, electromyographic signals, and basic reference signals. After training, a model with stronger generalization ability and stronger recognition ability for complex motion patterns is obtained, thereby improving the accuracy of human motion intention recognition based on sEMG technology and providing important support for the realization of human–machine fusion-embodied intelligence of exoskeleton robots.

In the context of the rapid development of artificial intelligence, the future sEMG-based human movement intention recognition technology will make breakthroughs in the following three aspects: improving the accuracy of sEMG signal analysis, adaptive modeling of different individuals, and embodied intelligence of human–machine collaboration. The fusion of artificial intelligence and multi-information will bring new ideas to the development of exoskeleton technology, improve its applicability in different environments and individuals, and achieve a more natural control strategy and control accuracy that is more in line with the physiological characteristics of the human body.

## Figures and Tables

**Table 1:** Innovative development of motion intention recognition techniques and applications in exoskeleton robots.

Author	Domain	Innovation
Yue Zheng et al. [60]	Artificial intelligence, collaborative robots	A new concept of human–robot intelligent collaboration is proposed combining human–machine interface technology and artificial intelligence so that they can complement each other’s strengths and work together to produce a more powerful intelligent form.
Liangmin Wei et al. [61]	Artificial intelligence, exoskeleton robot, Human–computer interaction	Innovatively combines artificial intelligence technology with traditional exoskeleton robot design; this technology will make it possible to provide personalized exoskeleton robot rehabilitation treatment plans for patients with paralysis, stroke, etc.
Fengxue Zhu et al. [62]	Rehabilitation robot, iterative learning control	Aiming at the nonlinear and uncertain problems caused by patient spasm disturbance in the trajectory tracking control process of upper-limb rehabilitation robots, a nonlinear iterative learning control algorithm was proposed for optimization.
Yali Liu et al. [63]	Exoskeleton robot, human biomechanics, comprehensive evaluation model	A set of exoskeleton assistance performance testing and evaluation systems was proposed and constructed to guide the research and development, product iteration, and actual use of exoskeleton robots in military, medical, and industrial fields, which has important theoretical innovation and practical significance.
Zhuangqun Song et al. [64]	Human motion intention recognition, follow-up lower extremity exoskeleton rehabilitation robot	In order to improve the support and following capabilities of the exoskeleton robot during use, a following tracking control strategy based on a dual radial basis function neural network adaptive sliding mode controller is proposed, which can accurately follow the motion trajectory and show better gait tracking performance than the traditional PID control algorithm.
Xiaoyun Wang et al. [65]	Motion intention recognition, adaptive control, active rehabilitation training	An adaptive admittance control scheme for lower-limb rehabilitation exoskeleton robots is proposed. The admittance model is used to ensure the compliant motion of the exoskeleton. NDO is used to estimate the interaction torque between human and exoskeleton robots in real time, which enhances the natural coupling and safety of human–machine interaction.
Seulki, K. et al. [66]	Exoskeleton, surface electromyography (sEMG)	A control algorithm using sEMG technology and torque sensor fusion is proposed to detect and compensate for unexpected interference factors caused by the collision between the exoskeleton and the surrounding environment, significantly improving the safety and interactivity of human–machine augmented robot equipment, providing technical support for the subsequent development of safer exoskeleton systems.
Xiao Feiyun et al. [67]	sEMG, hand/wrist exoskeleton, motion intention recognition	Aiming at accurately identifying and monitoring various hand movements using sEMG technology, an innovative hand exoskeleton structure design and signal processing algorithm are proposed to ensure that the exoskeleton can respond to the patient’s movement intention in a timely manner, thereby improving the efficiency and effectiveness of rehabilitation training.

**Table 2:** Innovative development of exoskeleton robots based on surface electromyography signal technology.

Author	Domain	Innovation
Jun Le et al. [68]	Surface electromyographic signal, exoskeleton upper-limb rehabilitation robot	A front-end acquisition and signal processing circuit based on surface electromyography signal technology is proposed. Filtering, shielding, isolation, and other measures not only solve the signal interference problem in the process of surface electromyography signal acquisition but also promote the application of SEMG technology in exoskeleton robots.
Hemiao Niu et al. [69]	Exoskeleton, surface electromyography, intention recognition	Aiming at the spatiotemporal differences and nonlinear dynamic characteristics of sEMG signals, a motion intention perception model based on a multi-scale convolutional neural network (CNN) is proposed to improve the feature expression richness and accuracy of sEMG signals.
Song Zhang et al. [70]	Intelligent rehabilitation, surface electromyography signal, intention recognition	It is proposed to apply the intelligent rehabilitation technology of surface electromyography (sEMG) signals to support quantitative diagnosis and objective evaluation of rehabilitation efficacy and to assist rehabilitation-type exoskeleton robots to achieve a safe and natural human–computer interaction experience.
Peishang Chang et al. [71]	Surface electromyographic signal, exoskeleton, BP neural network	A joint angle prediction method based on surface electromyographic signals (sEMG) is proposed. The BP neural network controller is used for joint angle prediction. Due to the non-invasiveness and real-time nature of sEMG technology, it can accurately predict joint angles and provide reference signals for the control of exoskeletons.
Jiyuan Song et al. [72]	Surface electromyography, movement intention, exoskeleton	In response to the need for the exoskeleton to quickly identify the wearer’s motion pattern in the hybrid control mode, a feature parameter dataset for training classifiers was constructed, providing reliable technical support for the precise control of the exoskeleton system.
Shi Xin et al. [73]	Exoskeleton, sEMG sensors	A filtering method integrating wavelet packet transform and sliding window difference mean is proposed, which can effectively suppress the noise interference in sEMG signals and provide support for the application of exoskeleton robots in complex environments.
Qiming Liu et al. [74]	Gait recognition, surface electromyographic signal, exoskeletons	A gait feature recognition method based on sEMG technology was proposed. The variational mode decomposition (VMD) algorithm and Gram angular field (GAF) were used to convert sEMG signals into two-dimensional image data, which improved the recognition accuracy and robustness of motion information.
Hao Zhou et al. [75]	Surface electromyography, movement recognition, sEMG	A model that combines ResNet and multi-scale feature extraction is proposed. By extracting and fusing feature values at different scales, the motion recognition performance of the lower limb exoskeleton robot is significantly improved.

**Table 3:** Comparison of machine learning model algorithms.

Name	Model Algorithm	Innovation	Features	Problems Addressed	Classification Accuracy
Duan	Time-domain features + LDA	RMSR and AR model for feature extraction	RMSR, AR	Impact of force variations on sEMG signals	91.70%
Naik	Modified ICA + PCA + LDA	Modified ICA weight matrix	sEMG and Cyberglove features	Classification of finger extension and flexion	90%
Qi	LDA + ELM	Characteristic Map Slope (CMS) extraction	CMS features	Optimization of temporal differences in sEMG pattern recognition	/
Benalcázar	k-Nearest Neighbor (kNN) + DTW	Real-time gesture recognition using Myo armband	EMG signal features	Real-time gesture recognition	89.50%
Narayan	k-Nearest Neighbor (kNN)	Time-frequency domain (TFD) features	FD features, TFD features	sEMG signal classification	95.5% (TFD), 89% (FD)
Bergil	k-Means Clustering + k-Nearest Neighbor (k-NN)	Four-layer symmetric wavelet transform for feature extraction	EMG signal features	Detection of six basic hand movements	86.33–100%
Nazemi	MLP, LDA, LS-SVM	Comprehensive evaluation of multiple feature combinations	Eight time-domain features	Recognition of 52 hand postures and gestures	96.34% (MLP)
Chen	MKL-SVM	Combination of three types of features	Time-domain features, ACCC, SPM	Digital gesture recognition using a 4-channel wireless sEMG system	97.93% (3F)
Fatimah	FDM + Multiple classifiers	Using FDM to decompose sEMG signals	Entropy, kurtosis, and L1 norm of FIBFs	Hand motion recognition	99.49% (UCI), 93.53% (NinaPro DB5)
Xue	Tensor decomposition	Tucker tensor decomposition for feature extraction	Three-dimensional tensor generated by wavelet transform	Gesture recognition	96.43%
Pourmokhtari	kNN	Single-channel EMG analysis	Max, Min, MAV, RMS, SSI	Finger movement classification	91.0–96.0%

**Table 4:** Comparison of deep learning model algorithms.

Name	Model Algorithm	Innovation	Features	Problem Solved	Classification Accuracy
Lin	GAN + CNN	Synthetic HD EMG signal simulation under interference conditions	Mean value of MEMG signals	Improving interference robustness in NMI applications	99.00%
Tam	CNN + Transfer Learning	Deep learning adaptive user EMG signal patterns	Unspecified	Real-time control strategy for prosthetic hands	93.43% PPV
Mohapatra	TFDDNN	Time-frequency domain deep learning network for automatic gesture recognition	Max, Min, MAV, RMS, SSI	Gesture recognition using multi-channel EMG sensors	92.73% and 80.33%
Jiang	Unspecified	None	Unspecified	Upper limb prosthetic control	Unspecified
Pourmokhtari	kNN	Single-channel EMG analysis	EMG signals	Finger movement classification	91.0–96.0%
Triwiyanto	Deep Learning	Recognition of multi-force variation gestures using amputees’ EMG signals	sEMG signals	Improving gesture recognition accuracy	0.92
Caraguay	Deep and Double Deep Q-Networks	Gesture classification and recognition using feedforward ANN and LSTM layers	Unspecified	Gesture classification and recognition	90.37–82.52%
Kong	RBF-based Sliding Mode Control	LSSVM-based joint angle prediction model	HOS-FD feature set	Upper limb rehabilitation training	Unspecified
Lv	SOM + RBF Network	Combining SOM feature selection and RBF network pattern classification	EMG signals	Hand motion intention recognition	0.9688
Rajapriya	Deep Learning Algorithm	HOS-FD feature set combined with DNN	sEMG signals	Accuracy of hand motion classification	0.9916
Rehman	CNN	Direct use of raw EMG signals as input to deep networks	EMG signals	Hand movement classification	Unspecified
Jiang	CNN	Time-spatial convolutional structure for feature extraction	sEMG signals	Shoulder muscle activation pattern recognition	0.9757
Simão	RNN, LSTM	Improving online gesture classification performance using LSTM-based dynamic models	EMG signals	Online gesture classification	Unspecified

**Table 5:** Comparison of machine learning, deep learning, and hybrid approaches.

Comparison Dimension	Traditional Machine Learning (ML)	Deep Learning	Hybrid Approach
Feature Extraction	Relies on manual feature engineering	Automatically learns hierarchical features	Combines manual and automated features
Data Requirements	Can train with small-scale data	Requires massive labeled datasets	Medium-scale data, partially relying on pre-trained models
Computational Resources	Runs on CPU, low computational cost	Requires GPU/TPU, high computational cost	Moderate resources (DL part fine-tunable, ML part lightweight)
